# Changes of borderline symptoms, personality functioning, and dysfunctional personality traits after dialectical-behavioral therapy in borderline personality disorder

**DOI:** 10.1007/s00406-026-02215-z

**Published:** 2026-05-08

**Authors:** Julia I. Kunz, Antonia Zwick, Jennifer J. Boll, Johannes Wolf, Judith Meinhardt, Sandrina Hüttner, Rafael Grigo, Richard Musil, Stephan Goerigk, Andrea Jobst, Frank Padberg, Matthias A. Reinhard

**Affiliations:** 1 Department of Psychiatry and Psychotherapy, LMU University Hospital, LMU Munich, Nussbaumstr. 7, 80336 Munich, Germany; 2 DZPG (German Center for Mental Health), Partner Site Munich-Augsburg, Munich, Germany; 3 Charlotte Fresenius Hochschule Munich, Infanteriestr. 11a, 80797 Munich, Germany; 4 Oberberg Fachklinik Bad Tölz, Buchener Str. 17, 83646 Bad Tölz, Germany

**Keywords:** Borderline personality disorder, Dysfunctional personality traits, Personality functioning, Dialectical-behavioral therapy, Alternative model for personality disorders

## Abstract

Borderline Personality Disorder (BPD) is a complex and debilitating condition characterized by limited treatment response and high dropout rates across treatments, even in leading therapeutic approaches, such as Dialectical-Behavioral Therapy (DBT). Given the heterogeneity of BPD symptomatology and the current shift toward dimensional assessments of personality disorders, this study investigates whether dimensional measures according to the DSM-5’s Alternative Model for Personality Disorders (AMPD) may serve as additional markers of treatment outcome and dropout. Eighty-five participants with BPD were recruited from a naturalistic inpatient DBT program. Associations between BPD symptoms (Borderline Symptom List, Borderline Personality Disorder Severity Index), personality functioning (Level of Personality Functioning Scale – Brief Form 2.0), and dysfunctional personality traits (Personality Inventory for DSM-5 and ICD-11 – Brief Form Plus) were examined at baseline, as well as their sensitivity to change after DBT. At baseline, both personality functioning and dysfunctional personality traits, except antagonism, were associated with BPD symptoms (all *r* ≥ 0.4). After 10 weeks of DBT, parallel improvements were observed in personality functioning and dysfunctional personality traits, with significant reductions in negative affectivity and detachment. Elevated baseline levels of negative affectivity and psychoticism were associated with less BPD symptom change. DBT dropout was linked to higher impairment in negative affectivity and anankastia at admission. Personality functioning showed greater sensitivity to change throughout DBT compared to dysfunctional personality traits. These findings emphasize how AMPD may yield benefits beyond categorical BPD diagnoses, potentially contributing to more personalized and effective treatment planning and preventing dropouts.

## Introduction

Borderline Personality Disorder (BPD) is a complex and often debilitating mental health condition characterized by pervasive instability in emotions, interpersonal relationships, self-image, and behavior [1–3]. It affects approximately 1–2% of the general population, with prevalence rates reaching up to 20% in clinical settings [1]. Impulsive and self-destructive behaviors, including self-harm and suicidality, are common in clinical presentations of BPD and typically emerge in adolescence [4–6]. In addition, intense mood swings, chronic emptiness, heightened sensitivity to rejection, and high rates of psychiatric and medical comorbidities further impair social functioning and quality of life [7], underscoring the urgent need for effective interventions.

Psychotherapy, such as Dialectical-Behavioral Therapy (DBT) [8], is one of the primary treatments for BPD and recommended by international guidelines [9, 10]. DBT combines cognitive-behavioral techniques with a dialectical framework that recognizes the challenge of balancing acceptance and the need for change throughout therapy. The program emphasizes validating patients’ experiences, often related to past traumatic events, while simultaneously fostering behavioral change by practicing mindfulness, enhancing emotional regulation, distress tolerance, and interpersonal functioning [8, 11]. DBT has proven effective in numerous studies, including independent meta-analyses [12–15]. A Cochrane review further highlights its specific benefits in reducing BPD severity, self-harm, and increasing psychosocial functioning [16]. The effectiveness of highly structured inpatient DBT programs has also been demonstrated, including 12-week treatments [17–19], as well as shorter programs lasting as little as two weeks [20, 21]. However, response rates remain limited, and dropout rates average 28% across 40 RCTs [22], underscoring the importance of identifying predictors of both treatment success and attrition.

Across BPD treatment modalities, previous research has already identified factors such as weaker therapeutic alliance quality, increased hostility, group therapy exposure, low affect regulation skills, and decreases in mindfulness practice as key predictors of dropout [23–27]. While research on these process-related predictors underscores the importance of therapeutic context and patient engagement, dimensional models of PDs have been proposed as an additional diagnostic lens that moves beyond categorical diagnoses. The DSM-5’s Alternative Model for Personality Disorders (AMPD) [1] reflects this shift by conceptualizing PDs in terms of moderate to severe impairments in self- and interpersonal functioning (criterion A) and maladaptive personality traits (criterion B), thereby providing a more nuanced description of each PD [28, 29]. Criterion B of the AMPD defines five dysfunctional personality traits (negative affectivity, detachment, antagonism/dissociality, disinhibition, and psychoticism) to capture individual expressions of personality pathology. This slightly differs from the ICD-11’s operationalization, which adds a sixth anankastia trait domain, and omits psychoticism [30, 31]. Within ICD-11, a BPD pattern has been retained as the only categorical specifier, reflecting its clinical utility and high prevalence [32].

While previous research has examined cross-sectional associations with AMPD constructs [31, 33, 34], longitudinal data on the effects of psychotherapy on personality functioning (PF) and dysfunctional personality traits, as well as their sensitivity to change, are still emerging. Significant improvements in PF have been observed across various treatment modalities and settings, e.g., after an average of 13.5 weeks of integrative psychoanalytic therapy [35], and in outpatient PD treatment, where PF improved particularly in individuals with a BPD diagnosis (≈ 0.31 LPFS-BF points/month) [36]. Further, increases in PF and reductions in antagonism were reported following 8–12 weeks of integrative dynamic-relational psychotherapy, although changes in maladaptive traits did not differ from a no-treatment control group [37]. Cognitive-behavioral interventions have also been associated with reductions in negative affectivity, detachment, and disinhibition, which partly predicted decreases in depressive and anxiety symptoms, although dysfunctional personality traits did not consistently predict or moderate treatment outcomes [38, 39]. Additional evidence points to reductions in psychoticism among day-hospital patients after 3 to 12 months, particularly in those receiving interventions with cognitive-behavioral elements [40]. Collectively these findings suggest that PF may be more sensitive to psychotherapeutic change than dysfunctional personality traits and may influence treatment adherence [37]. At the same time, the inconsistent predictive value of maladaptive traits [38, 39] emphasizes the need to clarify how PF and traits relate to treatment outcomes and dropout in BPD [36].

Consequently, this naturalistic study investigates the effects of a 10-week inpatient DBT program on BPD symptoms, PF, and dysfunctional personality traits. Specifically, we addressed the following questions: (1) Are BPD symptoms significantly associated with PF and specific dysfunctional personality traits? (2) Are PF and dysfunctional personality traits sensitive to change during PD-specific psychotherapy, and does PF show greater sensitivity to change compared to dysfunctional personality traits? (3) How are these factors related to treatment outcome and dropout?

## Methods

### Sample

Participants aged 18 to 65 and with sufficient proficiency in German to understand all interviews and questionnaires were recruited at LMU University Hospital from a ward that is specialized in the treatment of BPD with an elective 10-week inpatient DBT program [8]. The study complied with the Declaration of Helsinki and was approved by the Ethics Board of the Faculty of Medicine, Ludwig-Maximilians-University Munich (#713 − 15, study registration DRKS00019821). To participate in the DBT program, patients needed a confirmed diagnosis of BPD or a combined PD with emotionally unstable traits, a BMI above 17, no acute substance use disorder (or at least six months of abstinence), no acute suicidality (the most recent suicide attempt must have been at least six months ago), and a stable housing situation. Applications to the program had to include previous medical records and prior clinical assessments, where available. Eligibility for the DBT program was determined during structured pre-admission clinical interviews, whereas inclusion in the present study occurred after program admission, at which point diagnoses were systematically confirmed using the Structured Clinical Interview for DSM-5 (SCID-5-CV and -PD) [41, 42], administered by experienced clinicians or supervised research assistants. Symptom assessment referred to the time periods specified by the SCID. From July 2020 to July 2024, attending physicians screened patients for eligibility, and all participants provided written informed consent before study entry. Exclusion criteria comprised acute suicidality, acute psychosis, pregnancy, somatic conditions requiring primary treatment, or a prior stay in the same psychotherapeutic program. All self-reported and clinician-rated measures were completed at baseline and again after the 10-week program.

### Procedure

DBT was provided as a 10-week inpatient, interdisciplinary, and multi-component program. It included weekly individual therapy (two 50-minute sessions), and skills training groups (two 100-minute sessions), covering mindfulness, emotional regulation, interpersonal effectiveness, self-worth, and distress tolerance: modules designed for the specific needs of BPD [8, 11, 43]. Patients participated in weekly occupational therapy (three 75-minute sessions, one 50-minute session), a physician-led basic group (50 min), a mindfulness group (two 25-minute sessions), physical therapy (50 min), and art therapy (75 min). They also attended one-on-one nurse encounters (25 min) and physician visits (two 25-minute sessions). The treatment primarily focused on psychotherapy. Psychopharmacological interventions were used only to manage psychiatric comorbidities, in line with current national and international guidelines [9, 10]. Accordingly, existing medication was kept stable throughout the treatment period.

### Measures

*BSL-23*: The Short Version of the Borderline Symptom List (BSL-23) [44] was used for self-rating the severity of BPD symptoms over the past seven days. Scores can be categorized into six severity levels: none to low (≤ 0.28), mild (≤ 1.07), moderate (≤ 1.87), high (≤ 2.67), very high (≤ 3.47), and extreme (≤ 4) [45]. Psychometric evaluations including the German version of the BSL-23 show high internal consistency (α ≥ 0.93), high test-retest reliability (*r* = 0.84), moderate to high validity and good reliability in diagnosing BPD [44–46]. Internal consistency in our sample was high before (α = 0.91) and after DBT (α = 0.94).

*BPDSI-IV*: BPD symptoms were also assessed with the Borderline Personality Disorder Severity Index – Version IV (BPDSI-IV) [47–49], which evaluates BPD symptoms over the last three months. Interviews were conducted in strict adherence to the manual, ensuring that post-therapy assessments covered the entire 10-week DBT program as well as the two weeks preceding treatment. A German validation study established a cutoff of 21.3 or higher for clinically relevant BPD symptomatology [50]. The BPDSI-IV has high internal consistency across samples (α ≥ 0.85), high validity [48, 51] and shows high interrater reliability [47]. Internal consistency in our sample was high before (α = 0.88) and after DBT (α = 0.86).

*LPFS-BF 2.0*: PF was self-evaluated using the German version of the Level of Personality Functioning Scale – Brief Form 2.0 (LPFS-BF 2.0) [52]. The LPFS-BF 2.0 comprises 12 items, each rated on a 4-point Likert scale, ranging from (1) “very untrue” to (4) “completely true”, with higher scores indicating poorer PF. The total score, derived from the sum of the 12 items, ranges from 12 to 48. Six items assess self-functioning and six assess interpersonal functioning. The German version of the LPFS-BF 2.0 has demonstrated strong reliability and convergent validity [53], with McDonald’s ω ≥ 0.83 for reliability and *r* ≥ 0.72 for convergent validity across both domains [52]. Internal consistency in our sample was good before (α = 0.74) and after treatment (α = 0.86).

*PID5BF+*: Criterion B personality traits were self-rated with the Personality Inventory for DSM-5–Brief Form Plus (PID5BF+) [54]. To align with both DSM-5 Section III and ICD-11 trait models, an ant colony optimization algorithm reduced the original 220 PID-5 items [55] to 34 items, hierarchy-clustered into six higher-order maladaptive trait domains including anankastia, and 17 facets [54]. All items are rated on a 4-step response scale from (0) “very untrue” to (3) “very true”, with higher scores indicating higher symptom severity. Domain scores are calculated for Negative Affectivity, Detachment, Antagonism, Disinhibition, Psychoticism, and Anankastia, along with a total PID5BF+ score. Previous studies showed acceptable internal consistencies (α ≥ 0.70) for all five scales with substantial positive correlations within domains and an acceptable model fit via confirmatory and exploratory factor analyses [56–58]. However, all domains except Psychoticism showed inadequate convergent or discriminant validity for one to two items per scale [58]. In the present sample, internal consistency for the total score was high before (α = 0.84) and after DBT (α = 0.87). On a subscale level, overall acceptable to good internal consistencies were found for Negative Affectivity (α_before_ = 0.75, α_after_ = 0.81), Detachment (α_before_ = 0.64, α_after_ = 0.77), Antagonism (α_both_ = 0.78), Disinhibition (α_before_ = 0.72, α_after_ = 0.52), Psychoticism (α_before_ = 0.68, α_after_ = 0.76), and Anankastia (α_before_ = 0.61, α_after_ = 0.67).

### Data analysis

Analyses were performed using R version 4.5.0 (fixed significance threshold of α = 0.05). Pearson correlations between self- and clinician-rated BPD symptoms (BSL-23, BPDSI-IV) and both PF (LPFS-BF 2.0) and dysfunctional personality traits (PID5BF+) were computed for the baseline sample (*n* = 85). To examine sensitivity to change after 10 weeks of DBT, paired-samples t-tests were conducted for participants who completed the program (*n* = 53). Individual-level change in BPD symptom severity was evaluated using Reliable Change Indices (RCI) [59] for the BSL-23 and BPDSI-IV, based on baseline and 10-week follow-up scores. The RCI determines whether observed change exceeds what could be attributed to measurement error. RCI thresholds were computed using baseline standard deviations and estimated reliability coefficients of 0.97 for BSL-23 [60] and 0.93 for BPDSI-IV [47]. Since both measures are considered sensitive to temporal fluctuations, Cronbach’s Alpha was used as a proxy for reliability. Participants were classified as having reliably improved (RCI > 1.96), reliably deteriorated (RCI < -1.96) or not reliably changed (|RCI| ≤ 1.96). Finally, Pearson correlations were computed between baseline PF and dysfunctional personality traits and change scores in BSL-23 and BPDSI-IV to explore whether individual differences in AMPD-related variables were associated with symptom improvement during DBT. To control for Type I error inflation, p-values were adjusted using the false-discovery rate procedure [61].

## Results

### Demographics and clinical characteristics

In total, *n* = 89 DBT patients were recruited (female: 75.3%, male: 16.9%; other: 7.9; age: 26.6 [SD 8.3]; education in years: 14.0 [SD 3.1]). Baseline variables (including BSL-23) were available for *n* = 85 patients (see Table 1). In addition to a diagnosis of BPD, 29.4% of participants had comorbid PDs (24.7% with two PDs including BPD, 4.7% with three PDs including BPD). Among those with comorbid PDs, the most common were avoidant (27.1%), and obsessive-compulsive (8.2%). Other comorbidities were also common, particularly including major depressive disorder (78.8%) or persistent depressive disorder (49.4%), substance use disorders (31.7%), panic disorder (34.1%), agoraphobia (9.4%), social anxiety disorder (30.6%), PTSD (44.7%), binge eating disorder (20.0%), and somatic symptom disorder (15.3%). Please note that although substance abuse within the past 12 months was identified in this sample (as assessed by the SCID-5-CV), participants were only eligible for inclusion provided that no acute substance abuse was present at admission or that they had been abstinent for at least six months.

Out of 85 patients who provided baseline data, follow-up data after 10-weeks were available for *n* = 53 patients. *N* = 8 patients (9.4%) discontinued treatment, and *n* = 24 (28.2%) did not complete the 10-week assessments.

**Table 1:** Descriptive characteristics at baseline

	Total sample *n* = 85	DBT completers *n* = 53	Study dropouts *n* = 32	t_(81)_ or χ²	*p*
Age in years (SD)	26.56 (8.33)	27.74 (9.11)	24.48 (6.34)	1.74	0.087
Gender *(%)*				6.71	0.082
Female	66 (77.6%)	38 (71.7%)	28 (87.5%)		
Male	13 (15.3%)	12 (22.6%)	1 (3.1%)		
Other	6 (7.0%)	3 (5.8%)	3 (9.4%)		
Education in years	14.03 (3.11)	14.08 (3.16)	13.89 (3.08)	0.25	0.801
Living status *(%)*				9.75	0.045
Alone	18 (21.2%)	15 (28.3%)	3 (9.4%)		
With partner	15 (17.6%)	6 (11.3%)	9 (28.1%)		
With parents	19 (22.4%)	13 (24.5%)	6 (18.8%)		
Shared housing	12 (15.3%)	9 (17.0%)	4 (12.5%)		
Therapeutic housing	10 (11.8%)	9 (17.0%)	1 (3.1%)		
Age at onset	13.35 (4.38)	13.29 (4.14)	13.49 (4.96)	-0.17	0.866
BSL-23	2.07 (3.11)	1.89 (0.76)	2.38 (0.63)	-3.08	0.003
BPDSI-IV	27.3 (9.55)	25.65 (9.)	29.81 (9.10)	-1.52	0.132
Dimensional BPD score (SCID-5-PD)	13.53 (12.0)	14.1 (14.03)	12.05 (3.23)	0.67	0.508
LPFS-BF 2.0	34.84 (5.21)	34.42 (5.58)	35.6 (4.84)	-0.99	0.323
PID5BF+ Total	1.41 (0.38)	1.33 (0.40)	1.54 (0.31)	-2.47	0.016
Negative Affectivity	2.07 (0.59)	1.94 (0.56)	2.31 (0.57)	-2.82	0.006
Detachment	1.44 (0.58)	1.36 (0.61)	1.58 (0.51)	-1.71	0.091
Antagonism	0.79 (0.60)	0.72 (0.55)	0.90 (0.67)	-1.32	0.192
Disinhibition	1.57 (060)	1.55 (0.62)	1.59 (0.57)	-0.26	0.797
Psychoticism	1.09 (0.62)	1.03 (0.64)	1.18 (0.59)	-1.00	0.219
Anankastia	1.50 (0.66)	1.39 (0.64)	1.70 (0.64)	-2.10	0.039
Comorbid SCID-5-CV diagnosis (%)	*n* = 76 (100%)	*n* = 53 (100%)	*n* = 23 (100%)		
Major depressive disorder *(%)* (lifetime)	67 (78.8%)	48 (90.6%)	19 (59.4%)	1.57	0.210
Bipolar I disorder (lifetime)	5 (5.9%)	3 (5.7%)	2 (6.3%)	0.24	0.624
Bipolar II disorder ((lifetime)	1 (1.2%)	0 (0%)	1 (3.1%)	2.34	0.126
Substance abuse disorder (alcohol) (last 12 months)	20 (23.5%)	16 (30.2%)	4 (12.5%)	1.36	0.244
Substance abuse disorder (other) (last 12 months)	7 (8.2%)	4 (7.5%)	3 (9.4%)	0.58	0.447
Panic disorder (lifetime)	29 (34.1%)	22 (41.5%)	7 (21.9%)	0.83	0.361
Agoraphobia (last 6 months)	8 (9.4%)	5 (9.4%)	3 (9.4%)	0.29	0.591
Social anxiety disorder (last 6 months)	26 (30.6%)	18 (34.0%)	8 (25.0%)	0.04	0.842
Generalized anxiety disorder (last 6 months)	12 (14.1%)	7 (13.2%)	5 (15.6%)	1.05	0.306
Obsessive-compulsive disorder (last 4 weeks)	8 (9.4%)	4 (7.5%)	4 (12.5%)	2.06	0.151
PTSD (current)	38 (44.7%)	24 (45.3%)	14 (43.8%)	1.01	0.315
Anorexia nervosa ((lifetime)	7 (8.2%)	3 (5.7%)	4 (12.5%)	3.15	0.076
Bulimia nervosa (lifetime)	10 (11.8%)	6 (11.3%)	4 (12.5%)	0.77	0.381
Binge eating disorder (lifetime)	17 (20.0%)	13 (24.5%)	4 (12.5%)	0.26	0.613
Somatic symptom disorder (lifetime)	13 (15.3%)	11 (20.8%)	2 (6.3%)	1.38	0.240
Comorbid SCID-5-PD diagnoses (%)	25 (29.41%)	15 (28.30%)	10 (31.25%)	2.86	0.239
Psychiatric medication *(%)*	52 (61.17%)	32 (60.38%)	20 (62.5%)	12.26	0.092
Previous psychotherapeutic treatment *(%)*	64 (75.29%)	44 (83.02%)	19 (59.38%)	7.19	0.303
Suicide attempts in the past	2.07 (2.72)	1.67 (2.24)	2.94 (3.42)	-1.92	0.058

Note: *DBT* Dialectical Behavior Therapy, *BSL-23* Borderline Symptom List, *BPDSI-IV* Borderline Personality Disorder Severity Index, *SCID-5-PD* Structured-Clinical Interview for DSM-5 Personality Disorders, LPFS-BF 2.0 = Level of Personality Functioning Scale – Brief Form 2.0, PID5BF + = Personality Inventory for DSM-5 and ICD-11– Brief Form Plus, *SCID-5-CV* Structured-Clinical Interview for DSM-5 Clinical Version, PTSD = Post-Traumatic Stress Disorder, BPD = Borderline Personality Disorder. Please note that diagnostic time frames correspond to those defined in the SCID-5-CV (e.g., 12-months vs. lifetime assessments depending on the disorder)

## Baseline associations of BPD symptoms with AMPD measures

At baseline, self-reported BPD symptoms showed significant correlations with PF and dysfunctional personality traits, including negative affectivity, detachment, disinhibition, psychoticism, and anankastia (see Table 2). Antagonism showed a trend towards a significant correlation with self-reported BPD symptoms. The association of dysfunctional personality traits (PID5BF+ total) with BSL-23 was significantly stronger compared to PF (LPFS-BF 2.0) with BSL-23 (*z* = 2.67, *p* = 0.004). Regarding clinician-rated BPD symptoms, both PF and dysfunctional personality traits correlated with BPDSI-IV (no significant difference: *z* = 1.12, *p* = 0.12). All specific trait subscales correlated significantly with clinician-rated BPD symptoms (see Table 2).

**Table 2:** Correlation of self-reported and clinician-rated borderline symptoms with personality functioning, and dysfunctional personality traits at baseline (*n* = 85)

	BSL-23			BPDSI-IV		
	*r*	*p*	*p*_FDR_	*r*	*p*	*p*_FDR_
BSL-23	−			0.57	< 0.001	0.001
BPDSI-IV	0.57	< 0.001	0.001	-		
LPFS-BF 2.0	0.47	< 0.001	0.001	0.47	< 0.001	0.001
PID5BF+	0.65	< 0.001	0.001	0.56	< 0.001	0.001
Negative Affectivity	0.61	< 0.001	0.001	0.44	< 0.001	0.001
Detachment	0.40	< 0.001	0.001	0.28	0.020	0.023
Antagonism	0.20	0.070	0.070	0.27	0.020	0.023
Disinhibition	0.39	< 0.001	0.001	0.47	< 0.001	0.001
Psychoticism	0.47	< 0.001	0.001	0.45	< 0.001	0.001
Anankastia	0.36	< 0.001	0.001	0.24	0.04	0.042

Note: *BSL-23* Borderline Symptom List, *BPDSI-IV* Borderline Personality Disorder Severity Index, *LPFS-BF 2.0* Level of Personality Functioning Scale – Brief Form 2.0, PID5BF + = Personality Inventory for DSM-5 and ICD-11 – Brief Form Plus, *FDR* False Discovery Rate

### Change of BPD symptoms and AMPD measures after 10 weeks of DBT

**Fig. 1:**
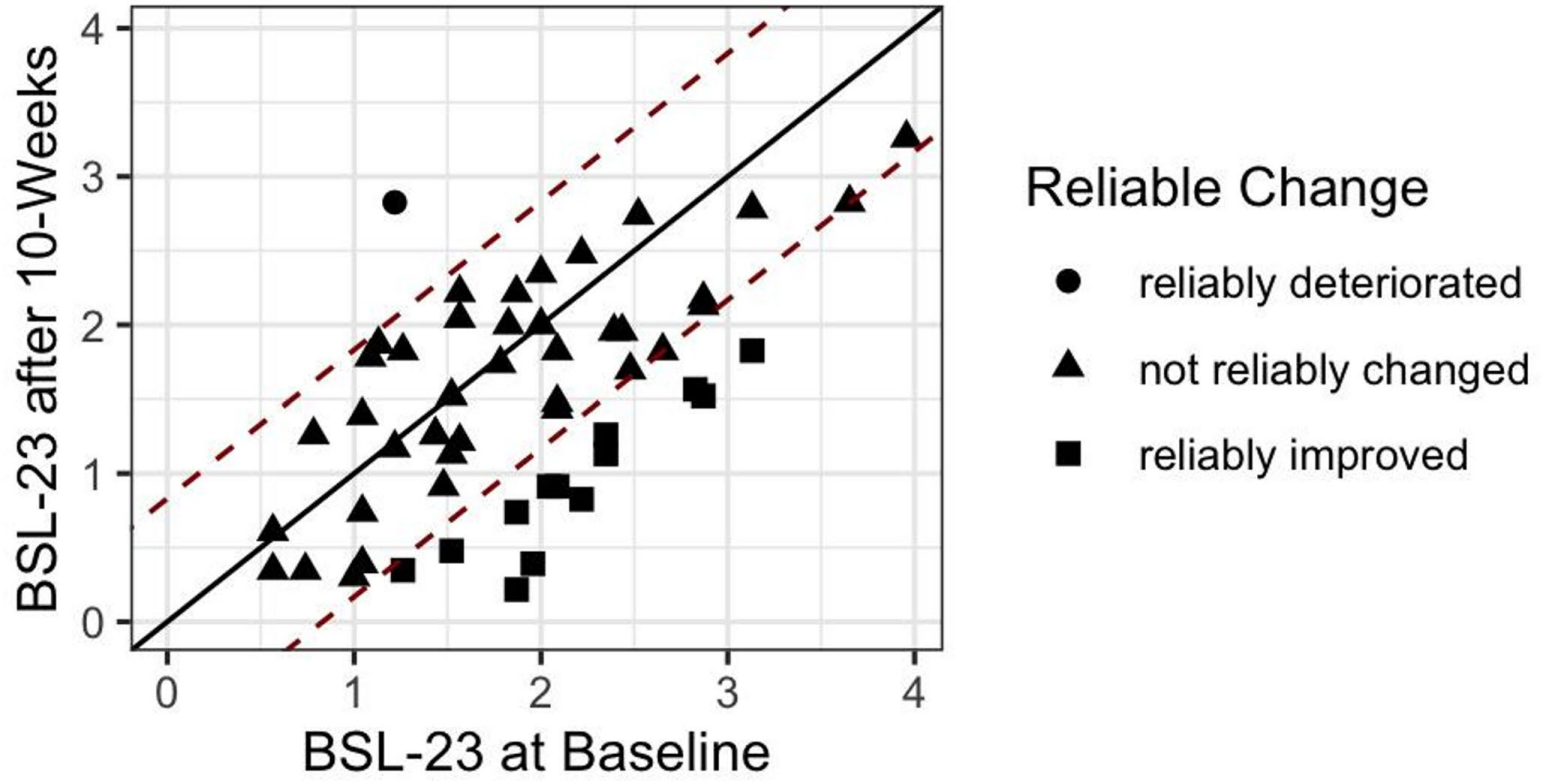
Reliable change of self-rated borderline symptomatology (BSL-23) after 10 weeks of Dialectical-Behavioral Therapy. *Description for accessibility.* The figure shows the change in self-rated borderline symptoms according to BSL-23 for patients undergoing Dialectical-Behavioral Therapy (DBT) over 10 weeks (divided in reliably deteriorated [13.2%], not reliably changed [34.0%], reliably improved [52.8%]). *Note.* All figures were generated in R version 4.5.0 using the base graphics and ggplot2 packages

**Fig. 2:**
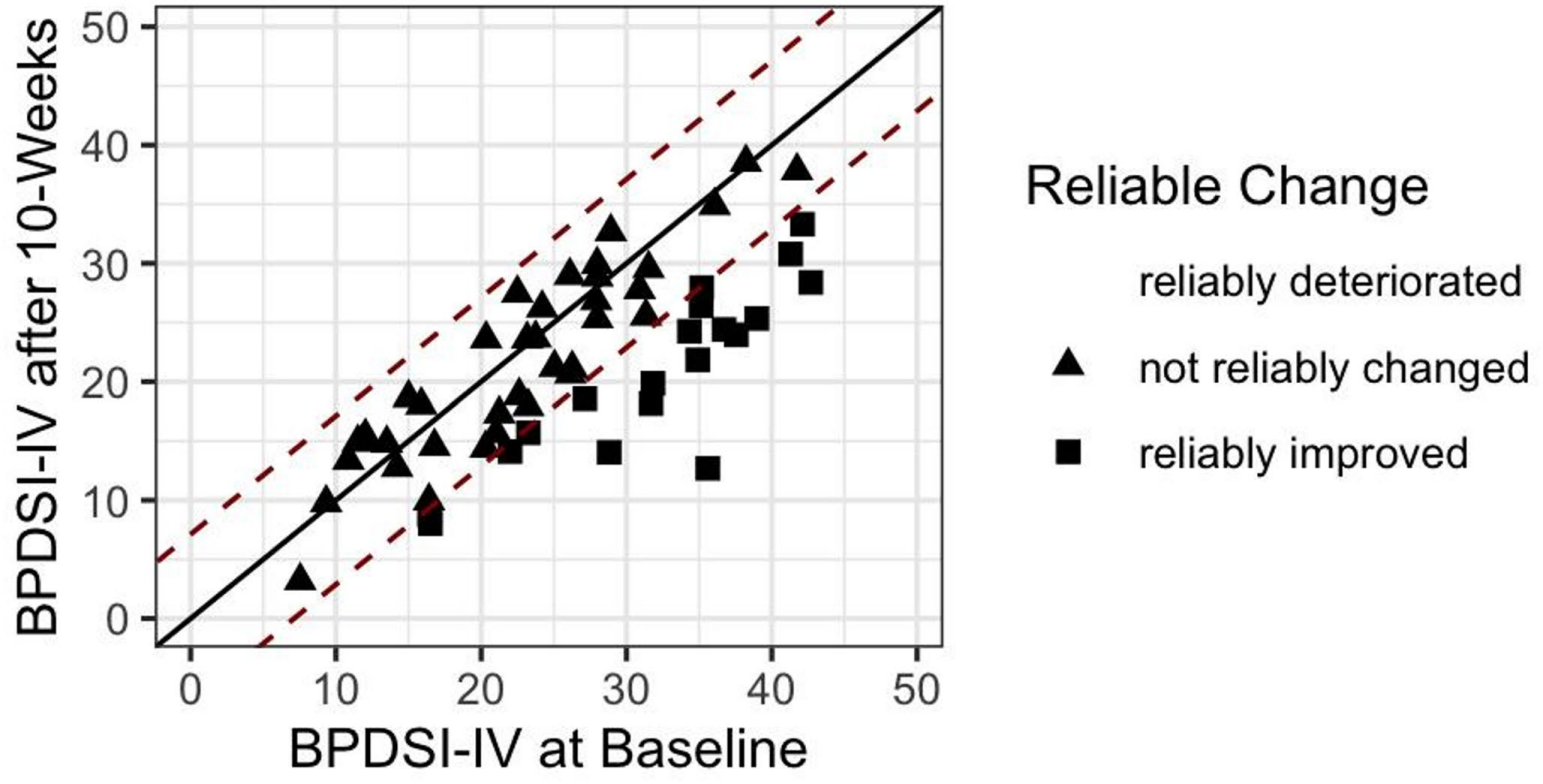
Reliable change of clinician-rated borderline symptomatology (BPDSI-IV) after 10 weeks of Dialectical-Behavioral Therapy. *Description for accessibility.* The figure shows the change in clinician-rated borderline symptoms according to BPDSI-IV for patients undergoing Dialectical-Behavioral Therapy (DBT) over 10 weeks (divided in reliably deteriorated [0.0%], not reliably changed [66.1%], reliably improved [33.9%]). *Note.* All figures were generated in R version 4.5.0 using the base graphics and ggplot2 packages

Self-rated BPD symptoms were significantly reduced after 10 weeks of DBT (*t*([52] = 4.1, *p* < 0.001) (see Table 3). The change of self-rated BSL-23 symptomatology reflected a change from high to moderate [45]. From the 53 completers, *n* = 28 (52.8%) showed a reliable improvement, whereas *n* = 7 (13.2%) reported a reliable deterioration, and *n* = 18 (34.0%) did not reliably change (see Fig. 1). In addition, clinician-rated BPD symptoms showed a significant reduction in total values (*t* [52] = 5.4, *p* < 0.001), with post-treatment scores approaching the clinical cutoff of 21.3 [50], reaching a mean of 21.5. Regarding the BPDSI-IV subscales, abandonment, interpersonal relationships, identity disturbance, impulsivity, parasuicidal behavior, and outbursts of anger showed significant improvements after 10 weeks of DBT (see Table 3). According to reliable change indices, 33.9% (*n* = 19) reliably improved, while none of the participants reliably deteriorated according to clinician-rated BPD symptoms (see Fig. 2).

Self-rated PF according to LPFS-BF 2.0 scores significantly improved after 10 weeks (see Table 3). In addition, the total score of the PID5BF+ showed a significant decrease indicating an overall drop of self-rated dysfunctional personality traits. The strongest improvement was observed for negative affectivity, followed by detachment. While psychoticism and disinhibition showed trends towards significant improvements, no significant change was observed for antagonism and anankastia (see Table 3). Changes in personality functioning were significantly stronger compared to total dysfunctional personality traits (*t*_*52*_ = 2.36, *p* = 0.022, *d* = 0.325 [0.05, 0.60]).

**Table 3:** Means (M) and standard deviations (SD) and the results of pre-post comparisons (*n* = 53) after 10-weeks of Dialectical-Behavioral Therapy

Measure	M (SD)					
	Before DBT	After DBT	t_(52)_	*p*	*p*_FDR_	d [CI95%]
BSL-23	1.89 (0.76)	1.50 (0.76)	4.10	< 0.001	< 0.001	0.56 [0.27, 0.85]
BPDSI-IV	25.65 (9.38)	21.46 (8.05)	5.16	< 0.001	< 0.001	0.74 [0.42, 1.03]
1. Abandonment	2.38 (1.37)	1.66 (0.99)	4.18	< 0.001	< 0.001	0.60 [0.29, 0.90]
2. Interpersonal relationships	1.93 (1.39)	1.38 (1.17)	3.35	0.002	0.005	0.48 [0.18, 0.77]
3. Identity disturbance	2.87 (2.05)	1.90 (1.55)	4.26	< 0.001	< 0.001	0.61 [0.30 091]
4. Impulsivity	1.44 (0.94)	0.98 (0.75)	3.51	< 0.001	< 0.001	0.50 [0.20, 0.80]
5. Parasuicidal behavior	1.31 (0.87)	1.00 (0.83)	3.03	0.004	0.008	0.43 [0.14, 0.72]
6. Affective instability	6.06 (2.17)	5.61 (2.30)	1.33	0.189	0.239	0.19 [-0.09, 0.47]
7. Emptiness	5.18 (2.57)	4.98 (2.51)	0.64	0.527	0.556	0.09 [-0.19, 0.37]
8. Outburst of anger	2.22 (1.66)	1.83 (1.40)	2.52	0.015	0.024	0.36 [0.07, 0.65]
9. Dissociation and paranoid ideation	2.27 (1.83)	2.13 (1.54)	0.94	0.353	0.295	0.13 [-0.15, 0.42]
LPFS-BF 2.0	34.42 (5.58)	29.57 (6.91)	6.09	< 0.001	< 0.001	0.84 [0.52, 1.15]
PID5BF+ Total	1.33 (0.40)	1.20 (0.44)	2.83	0.007	0.013	0.39 [0.11, 0.67]
Negative Affectivity	1.94 (0.56)	1.69 (0.65)	3.48	0.001	0.003	0.48 [0.19, 0.76]
Detachment	1.36 (0.61)	1.18 (0.65)	2.68	0.010	0.017	0.37 [0.09, 0.65]
Antagonism	0.72 (0.55)	0.66 (0.56)	1.19	0.241	0.286	0.16 [-0.11, 0.43]
Disinhibition	1.55 (0.62)	1.39 (0.81)	1.88	0.066	0.090	0.26 [-0.02, 0.53]
Psychoticism	1.03 (0.64)	0.90 (0.63)	1.93	0.059	0.086	0.26 [-0.01, 0.53]
Anankastia	1.39 (0.64)	1.35 (0.59)	0.52	0.603	0.603	0.07 [-0.20, 0.34]

Note: d effect size expressed in Cohen’s d; Cohen’s convention suggests interpretation of |0.2| as small effect, |0.5| as medium effect, and |0.8| as large effect. BSL-23: Borderline Symptom List, BPDSI-IV = Borderline Personality Disorder Severity Index, LPFS-BF 2.0 = Level of Personality Functioning Scale – Brief Form 2.0, PID5BF + = Personality Inventory for DSM-5 and ICD-11– Brief Form Plus, DBT = Dialectical-Behavioral Therapy

### AMPD associations with BPD symptom change and dropouts

The change scores of both self-rated and clinician-rated BPD symptomatology were negatively associated with PF and total dysfunctional personality traits at baseline (see Table 4). Negative affectivity, disinhibition, and psychoticism were negatively associated with changes in self-reported BPD symptoms. Psychoticism at baseline was further negatively associated with changes in clinician-rated BPD symptoms. Study dropouts self-reported significantly more BPD symptoms and dysfunctional personality traits, driven by significant differences in negative affectivity and anankastia, compared to DBT completers (see Table 1). Both groups also differed in their living situation. However, they did not differ in PF, age, gender, education in years, symptomatic age of onset, baseline medication, number of previous psychotherapeutic treatments, or suicide attempts in the past. In addition, no differences were observed in clinician-ratings according to BPDSI-IV or dimensional BPD assessment according to SCID-5-PD, nor in comorbid clinical or other PD diagnoses.

**Table 4:** Pearson correlations of changes in borderline symptoms with baseline personality functioning or dysfunctional personality traits

At baseline	Δ BSL-23			Δ BPDSI-IV		
	*r*	*p*	*p*_FDR_	*r*	*p*	*p*_FDR_
LPFS-BF 2.0	− 0.41	0.002	0.01	-0.35	0.008	0.02
PID5BF+ Total	− 0.44	< 0.001	0.01	-0.37	0.005	0.01
Negative Affectivity	− 0.40	0.003	0.01	-0.26	0.05	0.08
Detachment	−0.08	0.56	0.56	-0.21	0.12	0.14
Antagonism	− 0.27	0.06	0.08	-0.16	0.23	0.24
Disinhibition	− 0.37	0.007	0.02	-0.17	0.21	0.24
Psychoticism	− 0.41	0.002	0.01	-0.39	0.003	0.01
Anankastia	− 0.24	0.09	0.12	-0.29	0.03	0.05

Note: Δ = Delta, BSL-23 = Borderline Symptom List, BPDSI-IV = Borderline Personality Disorder Severity Index, LPFS-BF 2.0 = Level of Personality Functioning Scale- Brief Form 2.0, PID5BF + = Personality Inventory for DSM-5 and ICD-11– Brief Form Plus, FDR = False Discovery Rate

## Discussion

To our knowledge, this naturalistic study is among the first to examine changes in both criterion A PF and criterion B dysfunctional personality traits in individuals with BPD undergoing inpatient DBT. While prior research has largely focused on cross-sectional associations with AMPD constructs, longitudinal data on parallel changes in these dimensions during treatment remain scarce. To address this gap, we investigated associations of self- and clinician-rated BPD symptoms with the AMPD’s PF and dysfunctional personality traits (H1), and their change after 10 weeks of DBT, hypothesizing that PF would be more sensitive to change than dysfunctional personality traits (H2). Additionally, potential associations of baseline PF and dysfunctional personality traits on therapeutic outcome and dropout were explored (H3).

In our sample of patients with BPD, symptom severity was significantly associated with impairments in PF and dysfunctional personality traits at admission. Strong associations between dysfunctional personality traits and BPD symptoms have been reported before: In a study with university students, pensioners, and other volunteers, negative affectivity and disinhibition, followed by antagonism and psychoticism, accurately determined BPD [31]. Similarly, significant associations between PF, the PID5BF + M total score (a slightly modified “M” version of the PID5BF+), and self-reported BPD symptoms were observed in 131 individuals with PDs (mostly BPD with 55.7%) [33]. Consistent with these findings, we found significant associations with negative affectivity, disinhibition, and psychoticism [31], domains that may closely reflect core BPD features, such as impulsivity (disinhibition), fear of abandonment (negative affectivity), or dissociative/paranoid symptoms (psychoticism) [34]. Antagonism, reflecting a degree of hostility, was not prominently expressed in our sample, in line with the AMPD-based classification of core dysfunctional traits in BPD [1]. In contrast to prior research, we also found a significant association with anankastia, which may reflect an attempt to overregulate emotions and/or obsessive-compulsive features in our sample, as 8.2% of participants met the criteria for comorbid obsessive-compulsive PD, and 9.4% for obsessive-compulsive disorders.

Notably, dysfunctional personality traits showed stronger associations with BPD specific symptoms, particularly self-reported BSL-23 values, compared to PF. This finding may appear at odds with dimensional models that conceptualize BPD as primarily reflecting general impairments in self- and interpersonal PF [62]. The moderate to strong associations observed between both dysfunctional personality traits and PF with BPD symptoms in the present study rather suggest that BPD encompasses both general personality dysfunction as well as trait-based symptom expression. Moreover, the stronger association with dysfunctional personality traits may reflect the more behaviorally and affectively anchored nature of the BSL-23, BPDSI-IV, and PID5BF+ measures compared to the LPFS, which primarily assesses underlying capacities rather than specific behaviors. Overall, the broad overlap between AMPD severity and trait domains with BPD symptoms supports the notion that BPD features encompass a wide range of impairment relevant to general personality pathology [63].

Additionally, significant decreases in self- and clinician-rated BPD symptoms were observed after 10 weeks of structured inpatient DBT, particularly regarding abandonment, interpersonal relationships, identity disturbance, impulsivity, parasuicidal behavior, and outbursts of anger. Affective instability, feelings of emptiness as well as dissociative and paranoid ideations were not significantly reduced during the inpatient treatment period. This is in line with previous research confirming the general efficacy of DBT in reducing core BPD symptoms, particularly suicidality and parasuicidal-self-injurious behavior, with moderate to large effect sizes [12, 13, 15]. However, our findings also reflect limitations observed across longitudinal and meta-analytic studies, which suggest that affective instability, dissociation, identity disturbance, and chronic feelings of emptiness are less responsive to DBT or show only limited long-term improvement [64–66]. Recent randomized controlled trials further indicate that both DBT and Schema Therapy reduce BPD symptoms, although differences between treatments are not significant and some emotional and interpersonal symptoms persist [67, 68].

Consistent with previous finding, both criterion A and B showed sensitivitiy to change [38–40], and in line with our hypothesis, PF exhibited a stronger change compared to dysfunctional personality traits [37]. Particularly negative affectivity was significantly reduced, which describes a tendency toward intense and frequent experiences of negative emotions and affects as well as their behavioral and interpersonal manifestations, including separation anxiety, perseveration, mistrust, and the absence of restricted affectivity [1]. This effect may be attributed to DBT’s focus on emotional regulation strategies, i.e., with skills trainings, behavioral analyses, or its overall dialectical-therapeutic approach. By strengthening interpersonal competencies, DBT may also reduce social and emotional distance, potentially mediating the observed decrease in detachment (the tendency to withdraw from social interactions and emotional experiences [1]).

Notably, the significant change in negative affectivity contrasts with the relative stability of BPD criterion 6 affective instability as assessed by the BPDSI-IV, which describes frequent, rapid, and reactive fluctuations in mood over short periods of time [1]. However, affective instability in the BPDSI-IV is clinician-rated and refers to the frequency over the preceding three months, largely overlapping with the inpatient treatment of 10 weeks and may therefore mask short-term changes. Negative affectivity is based on self-report items without a strictly defined retrospective time window, potentially rendering it more sensitive to gradual change or biased by state-dependent factors [69]. Moreover, although conceptually related, the constructs only partially overlap. While negative affectivity reflects a broader tendency toward experiencing negative emotions, affective instability emphasizes reactive fluctuations. DBT skills might preferentially lead to a reduction in more chronic and intense negative emotionality rather than short-term affective reactivity. Yet these interpretations remain highly speculative given the naturalistic study design.

Disinhibition and psychoticism showed trends toward statistical significance, possibly reflecting improvements through enhanced impulse control (via emotion regulation skills) and reduced transient perceptual symptoms, such as dissociation or paranoid ideation, through mindfulness and reality testing. However, the lack of statistical robustness may also point to limitations of DBT in addressing the deeper underlying trait-features of these domains. For instance, DBT may not sufficiently target rigid cognitive distortions that contribute to psychoticism, nor offer sustained behavioral restructuring, which is needed to shift disinhibited tendencies. Moreover, a 10-week treatment duration may be insufficient to elicit measurable changes in broader personality traits, whereas domains such as negative affectivity and detachment may be more responsive to short-term interventions due to their closer alignment with core targets of DBT.

Finally, baseline PF and dysfunctional personality traits, including negative affectivity and psychoticism, were negatively associated with changes in self- and clinician-rated BPD symptoms, suggesting that individuals with elevated levels of these traits face greater challenges in engaging with DBT. Disinhibition at baseline was also negatively associated with changes in self-rated but not clinician-rated BPD symptoms, indicating that impulsivity/risk-taking may further hamper responsiveness. Contrasting findings have been reported in a large inpatient psychiatric sample (*n* = 804), predominantly comprising participants with mood, anxiety, or substance use disorders [70]. In that study, over a slightly shorter treatment duration (up to 8 weeks), higher PID-5-scores, particularly negative affectivity and detachment, were associated with greater therapeutic benefit. Notably, only 34% of participants were diagnosed with a PD compared to 100% in our sample. Additionally, treatment outcomes focused on depressive, anxiety, and somatic symptoms, as well as functional impairment, whereas the present study assessed changes in categorical BPD symptomatology. These differences suggest that elevated dysfunctional personality traits, while associated with higher symptom severity at admission, do not necessarily preclude positive treatment outcomes, depending on the outcome domains under investigation. Therapeutic engagement seems promising when pathological personality features are directly targeted, as in mentalization-based treatment [70].

According to our findings, dysfunctional personality traits did not appear to universally hinder psychotherapeutic response. However, they were associated with treatment dropout. Specifically, our dropouts reported significantly more self-rated BPD symptoms and more dysfunctional personality traits, particularly negative affectivity and anankastia. No differences in PF were observed. This is partially aligned with findings showing that participants in psychotherapy who did not complete a follow-up assessment exhibited higher negative affectivity, psychoticism, negative emotionality and lower extraversion compared to completers [37], although, in this case also exhibiting more impaired PF. These findings emphasize the potential clinical utility of assessing dysfunctional personality traits at the beginning of therapy to identify individuals at greater risk of dropout and to implement targeted strategies early on to monitor and sustain therapeutic engagement.

### Limitations

Several limitations should be considered. First, the recruitment of participants from a DBT program resulted in a naturalistic study design without a control group, such as one undergoing supportive psychotherapy or a waitlist condition, thereby limiting causal inference regarding changes in specific domains of PF or dysfunctional personality traits. Here, it remains unclear whether observed changes can be directly attributed to DBT, reflect differences in the time-sensitivity of these domains, or are influenced by moderating third variables. For instance, this is particularly relevant for changes in negative affectivity in relation to depressive symptoms, that share substantial conceptual overlap, as both involve frequent and intense experiences of negative mood, and emotional reactivity. Consequently, observed reductions in negative affectivity may partly reflect improvements in depressive symptomatology and/or antidepressive medication. Due to the naturalistic study design most patients received psychiatric medication and exhibited comorbid symptomatology. Although medication was generally kept stable and only adjusted if clinically necessary, we cannot determine with certainty the extent to which even slight changes in medication may have influenced observed outcomes. Second, patients were electively selected to the DBT program and had to meet specific inclusion criteria, resulting in a sample with lower baseline BPD symptom severity compared to other studies [48]. This may hamper generalizability of our findings to more clinically severe DBT inpatient or broader PD populations. An additional limitation of the RCI calculation is the use of Cronbach’s Alpha instead of the test-retest reliability, which may have produced a more liberal estimation of change by underestimating the standard error [71]. However, this deviation was deliberately chosen due to the time-sensitive nature of both BPD symptom measures, which naturally entails lower test-retest reliability. According to RCI most patients self-reported reliable improvements, whereas according to clinician-ratings 66.1% of participants did not reliably change. This discrepancy may reflect the BPDSI-IV’s three-month assessment period, which overlaps with admission and may mask early changes. Finally, due to the naturalistic study design, the correlative findings do not allow for directional or causal conclusions regarding the mechanisms underlying the observed changes and associations.

### Future research

Future research should replicate these findings in larger and more diverse samples, particularly including individuals with a wide range of PD symptom severity, from subclinical to severe levels. In addition, it is crucial to assess possible third variables, such as depressive symptoms and medication changes to allow a better understanding of the interplay between negative affectivity, (persistent) depressive symptoms, and personality dysfunction. Special attention should be given to BPD patterns and associated criterion A and B impairments, while also considering possible differences in the time-sensitivity of specific subdomains. As different BPD symptoms respond to DBT with varying degrees and timelines of change [12–15], changes in PF and dysfunctional traits should likewise be observed over extended periods. Particularly, since PDs are now understood to evolve over the course of an individual’s life and may be more adaptable than previously believed [72]. Head-to-head trials directly comparing treatment approaches are necessary to enable well-founded recommendations for this population [73]. Further, because dysfunctional personality traits at baseline may act both as a burden and as a motivator for therapeutic change, potential non-linear relationships as well as moderating aspects should be explored. In this context, previously reported indicators of dropouts, such as therapeutic alliance quality, hostility, group therapy exposure, affect regulation abilities, and mindfulness engagement [23–27], should also be examined in relation to baseline negative affectivity and psychoticism as well as overall PF to better identify individuals at risk of limited benefit from therapy. Of note, the therapeutic team was not informed about the results of PF and PID5BF+ at baseline, so this information did not influence the therapeutic process. Providing therapists with this information may enable a differential focus within DBT tailored to individual PD profiles.

Future research should further examine whether the stronger associations of dysfunctional personality traits with BPD measures replicate across independent samples. In a study with a mixed inpatient psychiatric sample (*n* = 966) using a bifactor model of DSM-IV PD criteria, it was found that BPD criteria loaded almost exclusively on a general ‘g’-factor of PD, with no residual BPD-specific factor, suggesting that BPD symptoms largely reflect core self- and interpersonal impairments rather than disorder-specific traits [62]. However, recent work suggests that general factors of PD (g-PD), psychopathology (‘p’-factor), and personality (GFP) primarily capture downstream functional impairments, such as social or occupational dysfunction, which arise secondary to traits and disorders rather than reflecting the traits themselves [74]. From this perspective, general factors index overall functional burden rather than the psychological processes giving rise to symptoms. Although this exceeds the scope of this study and cannot be adjudicated due the study’s design, the temporal and structural relationships between PF, dysfunctional personality traits, and BPD-specific symptoms warrant further investigation to disentangle whether impairments in PF represent core mechanisms of BPD or downstream consequences of dysfunctional personality traits and symptom expression.

Finally, given the heterogeneity within BPD, where meeting any five out of the nine diagnostic criteria yields up to 256 distinct symptom combinations [75], trait-based distinctions have been suggested to enhance our understanding of potential BPD subtypes [76]. Previous work has proposed three subtypes (internalizing, externalizing, mixed) [77, 78], which conceptually align with the AMPD’s and ICD-11’s dysfunctional personality traits. The internalizing presentation, characterized by high identity disturbance, or feelings of emptiness, may reflect high negative affectivity and/or anankastia. Externalizing individuals, characterized by more impulsivity and aggression, are more likely to exhibit disinhibition, negative affectivity, or dissociality [76, 78]. Mixed subtypes show a broad elevation across multiple traits, including negative affectivity, disinhibition, dissociality, and detachment [76]. Here, empirical studies in larger samples are needed to validate these conceptual distinctions and explore their clinical utility, particularly for differential treatment planning and therapy outcome.

## Conclusion

In conclusion, this study provides evidence for an interplay between BPD symptom change and negative affectivity and psychoticism, as well as for an association of dropout with negative affectivity and anankastia during 10 weeks of inpatient DBT. Consistent with previous research, criterion A showed higher sensitivity to change than criterion B in the context of DBT, although significant improvements were observed in both domains. These findings point towards a benefit of implementing individual trait patterns into clinical practice and treatment planning. A combined approach may offer advantages beyond the traditional BPD diagnosis alone, potentially informing the development of personalized therapeutic approaches and preventing dropout.

## Data Availability

The data that supports the findings of this study are available from the corresponding author upon reasonable request.
